# Green and Social Regulation of Second Hand Appliance Markets: the Case of Air Conditioners in the Philippines

**DOI:** 10.1007/s43615-022-00212-7

**Published:** 2022-09-17

**Authors:** Babette Never

**Affiliations:** IDOS German Institute of Development and Sustainability, Tulpenfeld 6, 53113 Bonn, Germany

**Keywords:** Air conditioners, Philippines, Energy efficiency, Climate change, Reuse, Repair

## Abstract

Second hand markets for appliances such as air conditioners are largely unregulated in many low and middle income countries this far. Energy and climate goals may require a speedy phase-out of old appliances, whereas material resource and social concerns may call for repairing and reusing air conditioners as long as possible. Demand for space cooling is soaring globally, increasing regulatory pressure. In middle income countries such as the Philippines, the market for second hand room air conditioners is sizeable. This study targets the question when and how to regulate the market for used air conditioners to balance green and social goals. It analyses the second hand market for air conditioners in Metro Manila, uncovering the general supply chain, business models and customers as well as energy efficiency, refrigerant and repair practices. The study draws on qualitative interviews with 10 experts and 29 retailers and technicians active in the semi-formal second hand market for air conditioners. Available information of lifecycle analyses is taken into account, but the focus of this contribution lies on social impacts of potential regulation. Overall, short-to-medium interventions in the second hand market are required to balance environmental and social goals that target the different players in the market: construction industry, brokers, retailers and technicians, customers and scrap dealers. Neither a complete ban nor delaying or foregoing regulation is advisable. Specific policy recommendations are derived.

## Introduction

Policymakers and technical cooperation agencies around the world tend to focus either on the active phase-in of new green technologies or on the end-of-life (e-waste) stage, neglecting the stage in between. The decisions if, when and how to speed up the phase-out of old appliances deserves more attention. In many low- and middle-income countries, large second hand markets for used appliances such as air conditioners (AC) exist. Apart from health and safety requirements and guidelines on e-waste that generally encourage reuse of technologies, more concrete regulations on used appliances such as ACs are missing in many low- and middle-income countries this far. From an energy and climate perspective, taking old ACs off the market as quickly as possible seems desirable [7]. However, a material resource and lifecycle lens may change the picture. Furthermore, social questions of affordability in low- and middle-income countries rather call for prolonged use of cheaper, repaired ACs. Managing this complexity requires political attention to growing second hand markets.

The demand for global space cooling and, correspondingly, the sales numbers of room ACs are soaring, especially in tropical middle-income countries such as the Philippines. Energy demand for space cooling is expected to triple globally till 2050 [18]. The room AC market in the Philippines was already sizeable with approx. 700,000 new units sold annually in 2016; it increased to approx. 900,000 units in 2018 [12, 31]. Growth trends in the room AC market are expected to continue strongly on the back of more home staying activities and a very limited online market, in spite of overall economic impacts of the COVID-19 pandemic.

These market dynamics present a challenge from an energy security perspective, but also from a climate change perspective. Global warming effects deriving from both electricity use of ACs (if non-renewable electricity source) and climate-harmful refrigerants in the AC are tremendous. Globally, energy efficient, climate-friendly cooling could save up to 8 years of global emissions at 2018 levels (IEA 2020). For the Philippines, cooling sector emissions are expected to rise from 24.7 MtCO_2_eq in 2017 to 44.6MtCO_2_eq in 2050 in a business as usual scenario [12].

In parallel, positive impacts of AC ownership and use on human development are fairly clear. Studies have found better health (sleep, disease control) and higher productivity in cooled environments [4, 20, 21]. A growing AC market is likely to have positive employment effects on local technician and service enterprises. Moreover, many livelihoods depend on formal and informal scrap dealing and recycling of e-waste, including ACs [1]. Therefore, an emphasis to forego the purchase of ACs should not be the primary political strategy in tropical middle-income countries, but rather a market transition to green ACs. Green ACs can be energy efficient and contain climate-friendly refrigerants such as R290.

In contrast, repairing, reusing and recycling appliances such as ACs can make sense from a lifecycle perspective and to support the move towards a circular economy [5, 17]. Additionally, socioeconomic benefits of repair services and trade of used goods in terms of employment and income need to be taken into account. Thus, the existence of a second hand market for ACs in low- and middle-income countries is not necessarily bad. The key question then becomes: *Under which conditions and how to regulate the second hand AC market to balance energy, climate, resource and social impacts?* This contribution aims to help answering this question with a social science analysis of the current second hand AC market in the national capital region of the Philippines, known as Metro Manila. It analyses prevalent business models, sales, prices and customers as well as energy efficiency practices and resource use in the market. Policy options for balancing environmental and social goals will be derived with an empirical focus on social goals and impacts of potential regulation on various stakeholders in the market.

While it is hardly possible to reliably quantify the size of the market for used or “second hand” room ACs in the Philippines, it is safe to assume a substantial market size that requires political attention. According to the Refrigeration and Air Conditioning Technician Association of the Philippines (RACTAP), there are approximately 5,000 AC technicians in Metro Manila alone, possibly more, as not all are registered with RACTAP. Many technicians are also retailers of ACs. A conservative estimate: if only half of these 5,000 retailer-technicians sold 20–30 used AC units per year, this would already amount to 50,000–75, 000 second hand ACs sold in Metro Manila alone. Extrapolated to all of the Philippines, the numbers of used ACs sold annually could likely reach 200, 000–300, 000, roughly a third of the market for new units. At the time of writing, the second hand AC market was unregulated apart from a general hazardous waste disposal requirement, health and safety requirements and a ban of the refrigerant R22 as of 2021. Regulations for electronic waste are currently being developed [1].

The remainder of this paper is structured as follows: The “Literature Review: Regulating Material Use vs Energy Efficiency and Carbon Footprint?” section summarizes existing regulatory approaches and reviews available lifecycle assessment and footprint literature, followed by a section on the methods employed in the study (“Methodology” section). The “Results and Discussion: Business Models in the Second Hand AC Market in Metro Manila” section presents and discusses the results on the AC market in Metro Manila, analysing business types and competition, sources of ACs and middlemen, AC types and prices, customers and their purchase motivations as well as insights on energy efficiency and refrigerant handling. Finally, policy recommendations for green and social regulation of the second hand AC market and conclusions for future research will be drawn.

## Literature Review: Regulating Material Use vs Energy Efficiency and Carbon Footprint?

The challenge in regulating the used AC market lies in, first, *finding most suitable combination of measures* to phase-out inefficient, climate-harmful devices without increasing material resource use and waste and without jeopardizing social livelihoods, and, second, in *finding the most suitable point in time for intervening* in the product lifecycle. This section summarizes the existing regulatory approaches to the reuse of appliances and end-of-life management as well as the technical information provided by the available lifecycle assessments (LCA) for ACs. From the latter, theoretical environmental policy options (green goals) can be derived. The empirical analysis in the remaining sections then focuses on the functioning of the second hand AC market and potential regulatory impacts on livelihoods (social goals).

Regarding the *most suitable combination of measures*, the specific balancing challenges for used ACs has not been completely recognized yet by policymakers. Two challenges for used electronic appliances have been identified in general [23]: First, the methodological question how to best assess the reusability of appliances in terms of the many environmental, social, legal, technical and cultural factors is still open. Second, comparative information and functionality tests are lacking. Consumers would need to be able to compare the functionality, energy efficiency and resource footprint of a used product to an available new product. Recyclers require more information on component structure and durability. However, economically feasible functionality tests of used appliances and labelling after testing are lacking.

In many countries, the reuse of appliances features in waste management regulations which are part of WEEE policy packages, for example, in the EU and China. As the reuse of white goods and private resales often happen without policy incentives and a range of product lifetime extension models exist already [10, 23], second hand markets do not necessarily require regulation for all types of goods. In the EU, reuse in WEEE policies is part of a broader recycling policy package. This emphasis on recycling may lead to a neglect of developing and implementing specific repair and reuse standards by member states [29]. Lu et al. [23] also argue that the ambiguity of terminology and insufficient appreciation of product/component reclamation over recycling hamper existing WEEE policy effectiveness in the EU. In China, in turn, reuse of appliances and components is popular without policy support [23]. To the author’s knowledge, there are no specific regulatory approaches aimed at reusing ACs, e.g. the time a used AC should remain in the market or the repair standards it has to fulfil. Table 1 provides an overview of the main regulatory approaches relevant for used ACs, even if these mostly target the end-of-life stage or electronic waste more generally, without specifically targeting ACs.

**Table 1:** Overview of main electronic waste and F-gas regulatory approaches relevant for ACs

Type of regulation	Policy objective	Scope/AC specified	Countries with this regulation in place (non-exhaustive)
Limiting quota of F-gases allowed to be sold (phase down)	Phase down of F-gases in line with the Montreal Protocol	F-gases used in any types of device, including air conditioners	European Union (EU)
Ban of F-gases in new equipment	Phase down of F-gases in line with the Montreal Protocol	F-gases used in any types of device, including air conditioners	EU
Ban of import of hazardous waste	Reduce amount of hazardous waste in line with Basel Convention	In some countries unclear whether used ACs with/without refrigerant are included	EU, China, India
Ban of import of used AC*	Reduce amount of e-waste and protect local producers	Specific for ACs	China, Ghana, Indonesia, Vietnam
Regulation of AC import	Control import process	Permit application required	Thailand, Philippines
Extended Producer Responsibility	Collection of instruments that make producers responsible for the (e.g. product-take back, deposit-refund systems, performance standards such as quotas for recycling or recommerializing)	Vast scope of general regulation, usually specified for single products. Japan specific ACs in their Home appliance recycling act (89% recommercialization ratio)	China, Japan, Taiwan
Other regulations of manufacturers and retailers	Obligations of appliance manufacturers and retailers to ensure proper waste treatment and efficient use of resources	The scope and degree of obligation and control vary between countries and products	EU, Japan, Singapore, Korea, Philippines
Specific regulation on safe disposal	Requires companies/individuals receiving, treating, transporting, or storing hazardous waste have to seek permission from the Pollution Control Board and bans the dumping of e-waste		Singapore, Philippines

*Other countries ban the import of used electronic waste without specifying ACs. Sources: [15, 27]. For a complete list of regulatory approaches, refer to these sources

As reuse of components and remanufacturing happens a lot more in low- and middle-income countries than in high-income, industrialized countries, a different combination of policy measures and managements systems may be required [23]. Furthermore, the current political focus on phasing-in new energy efficient appliances often comes with the expectation of a swift trickle-down effect, pushing old, inefficient appliances out of the market [25]. This process may, however, be much slower than policymakers expect, given that second hand markets present a blackbox at this stage.

Extended producer responsibility (EPR) schemes are being discussed and implemented in many low- and middle-income countries as a key approach for WEEE. However, the reduction of waste upstream by explicitly including quality standards for used goods and/or standard reuse procedures (preparation, dismantling, transfer, disposal, sales) presents a gap in most existing EPR schemes [6]. In the EU, general reuse standards laid out in the 2012 WEEE directive do not integrate well with EPR schemes [23]. Current take-back regulations of e-waste can be differentiated into those without reuse targets, aiming only at collection, and those with reuse targets, aiming at both collection and reuse [8]. Higher reuse targets may benefit consumers if there is competition in remanufacturing in a regulated market [8]. Additionally, the various actors involved in the reuse and end-of-life chain such as producer organisations and collectors may have opposing interests and lack communication, as first studies in European countries have shown [6]. In low- and middle-income countries, the functioning of the used AC markets, actor composition and interests as well as the current levels of repair, reuse, energy efficiency and climate impacts are still unclear. This makes the identification of regulatory starting points difficult.

In the Philippines, neither an EPR scheme nor standardized collection or reuse targets have been set at this stage. The import of second hand electronics requires a permission by the Department of Environment and Natural Resources (DENR), but the sector is overall not well regulated yet [34]. The Republic Act 6969 sets out regulations on the importation, manufacture, processing, handling, storage, transportation, sale, distribution, use and disposal of chemical substances and mixtures that present unreasonable risk and/or injury to human health or to the environment in accordance with national policies and international commitments. This includes refrigerants of ACs. Companies importing ACs and handling refrigerant disposal need to register with the DENR. Two specific administrative orders by DENR provide procedures for the handling of hazardous waste and WEEE. Household white goods shall be consolidated by Material Recovery Facilities; specific guidelines and precautionary measures for the registered facilities treating and disposing WEEE for environmentally sound management exist (DAO 2012–22). At the time of data collection for this study, the regulatory framework for e-waste, including ACs, was being re-evaluated and updated, including a potential combination with an F-Gas regulation and phase-out schedule in line with the Montreal Protocol. To the authors’ knowledge, no final new legislation or administrative orders have been published till the time of writing; depicting trends here would therefore amount to speculation. The government entities contacted during the course of this study were particularly interested in understanding the used AC market, being fully aware that current regulation does not reach this market (see the “Methodology” section). No specific, reliable data on the number of used, reused and disposed ACs in the Philippines is available at this stage.

For ACs, a combination of measures may be required that support the reuse of metals and non-hazardous components of the AC, but the phase-out of inefficient complete ACs or harmful parts such as chemicals and refrigerants. The latter requires a set-up of a collection system and management of ozone depleting substances (ODS) banks which are missing in many countries this far, including the Philippines [13]. LCA provide the technical background to guide a prioritization of measures for used ACs from an environmental perspective.

There are no LCA for residential ACs in the Philippines available that include repair and reuse, which are necessary to understand both first and second hand market impacts. Available LCA for other middle-income countries such as China, Indonesia and India have found that energy and climate impacts are highest during the use stage of the AC, not during manufacturing, transport or sales [14, 19, 35]. This is primarily due to the share of fossil fuels in the electricity mix, confirming the findings of LCA in the USA [22, 30]. Importantly, not only the user behaviour itself (e.g. active use duration, leaving the AC on when not in the room), but also the electricity required to keep the refrigerant cool, while the unit is idle contribute up to a third of the lifecycle emissions in hot countries [28]. In European countries, a switch to a renewable energy mix would decrease the global warming impact of household appliances, including ACs, by 25% [17]. However, this scenario would not reduce appliance use impact on ozone depleting substances, as these would continue to be used in ACs in such a single shift scenario.

Steel and copper have the greatest influence on environmental resource footprint during AC manufacturing [3]. For Indonesia, a country comparable to the Philippines in terms of electricity mix and market structure, Karkour et al. [19] confirm that the use stage of ACs has the highest global warming potential due to electricity use (90%), which are also higher than direct emissions from refrigerant leakage. Copper and nickel are found to be responsible for the largest share of resource consumption impacts (50%) and also have the highest price due to being more scarce than iron, for instance. Looking at AC parts in more detail, the compressor and the heat exchanger (copper and iron) make up the largest share of the resource footprint [19]. For a potential regulation of used ACs, these findings imply that energy and climate goals prevail over resource footprint concerns (unless copper and nickel become scarce).

To identify the *most suitable point in time* in the lifespan to intervene, assessments of the emissions and electricity consumed during AC manufacturing need to be balanced with prolonged lifetime impacts. As Nishijima [26] shows for Japan, shortening the life time of ACs by 1 year to maintain the carbon emission levels of 1990 requires a stricter strengthening of minimum energy performance standards (+ 20.6%) than setting incentives to prolong AC possession by 1 year (standards strengthened by + 17.8%)—with the same result. The choice of political measure depends on what the local AC industry can realistically achieve. Minimizing energy consumption requires more frequent replacement of residential central ACs over time than minimizing greenhouse gas emissions [7].

Additionally, the impacts of repair and replacement of parts as well as differences in electricity use intensity, especially for inverters, according to run time, temperature setting and cleanliness need to be taken into account. A more in-depth study of the amount of waste and recycling flows would be required, given that not all parts of an AC can be recycled and environmentally ideal waste and recycling systems may not (yet) exist. While Karkour et al. [19] recommend to recycle used units as quickly as possible, the identification of a concrete, suitable point in time for intervening in the lifecycle of used ACs is still not straightforward. Regarding environmental costs vs human benefits, the authors found that the share of materials costs are lowest during a 10-year lifespan (1–3% of costs only). The inverter-type AC has the highest benefits, mainly due its low impact in the use phase.

In sum, the current literature suggests that a combination of repair and reuse standards for used ACs, reuse targets and collection processes for precious metals such as copper and nickel as well as the management of ODS-banks would be useful. From an environmental perspective, prioritizing energy and climate goals means intervening in the used AC market to stop inefficient, repaired models to circulate for too long, i.e. more than 10 years, possibly even shorter. Limiting the lifetime of ACs (new and reused) may need to be strategically combined with tightening MEPS for ACs. These theoretical options now need to be put into the empirical context of the second hand AC market in Metro Manila. In addition to technical and environmental factors, questions of affordability and access to cooling as well as the livelihoods connected to the business need to be considered to derive a suitable regulatory approach.

## Methodology

The goal of this study is to assess options, potential impacts and acceptance of regulating the second hand AC market, with an empirical focus on socioeconomic aspects in the market. To assess these, quantitative modelling of environmental impacts or of general regulations on unknown market structures are hardly suitable. Rather, as a first step, a qualitative understanding of the market, its functioning, the stakeholders and their interests and interactions is required.

Conceptually, the analysis draws on studies on micro and small enterprise development and informal markets in developing countries (e.g. [2, 11, 16, 24], taking a broad business model lens. Key elements of such formal and semi-formal market analyses involving micro and small enterprises typically include an assessment of firm characteristics (e.g. business size, informality/registration and sales/turnover), entrepreneurial characteristics (e.g. education, skills) and the business environment (e.g. access to resources, business practices, competitive advantage). This analytical lens as well as the insights of LCA for ACs summarized in the previous section serve as starting points for the empirical analysis of the current second hand market for ACs in Metro Manila. Whereas a deeper technical assessment is not the core interest of this study, reported energy efficiency of the units found in the second hand market, energy saving considerations as well as repair and refrigerant-related practices by technicians and consumers will be taken into account. These indicate the general level of environmental impact of current practices.

Methodologically, this study takes an inductive, qualitative approach. A content analysis following Mayring (2003) was conducted on policies, background materials and qualitative interviews. In March 2020, 10 background interviews with experts, business organisations, international donor agencies and members of government were conducted in Metro Manila. The list of institutions interviewed is provided in the Annex. At the end of each interview, each expert interviewed was asked to recommend other people in the field to further discuss the matters (snowballing technique). Due to the outbreak of the COVID-19 pandemic in March 2021, complete saturation was not reached as three further expert interviews had to be cancelled (see Annex).

Subsequently, 29 semi-structured interviews with second hand retailers and technician-retailers were conducted throughout Metro Manila until end of November 2020. The background interviews served to understand the current state of information and the policymaker view on the topic. Policymakers were aware of some shop locations and rough prices for second hand units, but had no in-depth knowledge how the business and supply chain in the second hand market works. The role of energy efficiency as well as current repair practices and refrigerant leakage was also largely unknown, but deemed relevant for future policymaking. Additionally, the background interviews were used to refine the semi-structure for the interviews with retailers and technician-retailers.

The semi-structured interviews finally included questions on the business type and firm characteristics; sources and types of ACs sold; customers and sales; competitors, competitive advantage and market functioning; energy efficiency/energy saving awareness, refrigerant and repair practices (relating to both entrepreneur skills and customer interest). Finally, compliance with current regulations and opinions on potential regulation of the used AC market and acceptance of public or private intervention were sought, e.g. via an extended producer responsibility (EPR, explained in easy wording).

The interviews were partly conducted in English, partly in the local language Tagalog with the help of a local consultant, and translated to English. Given the sensitivity of some of the topics addressed (e.g. bribery, non-payment of taxes), we refrained from recording and took notes only. Respondents were assured that their answers remain anonymous; statements are therefore mainly summarized and interview quotes attributed to a randomized numbering of interviews. Interview notes were coded and analysed using atlas.ti.

## Results and Discussion: Business Models in the Second Hand AC Market in Metro Manila

### Business Types and Competition

The market for second hand ACs in Metro Manila is primarily situated in the semi-formal and informal sectors. Semi-formal sector here is defined as follows: The AC technician belongs to a larger, registered business, but sells second hand ACs informally; or the micro or small enterprise is registered, but does not pay all taxes; or it is only registered with the Department of Trade and Industry (DTI), but not the local government unit (LGU). Several fully formal, registered AC enterprises engage in the second hand market as well.

The businesses in the second hand market are micro and small enterprises, ranging from an individual technician to a small enterprise with 1–7 staff members. Staff are often family members, but also hired labourers or sub-contracted technicians for fixed time periods. Only one enterprise interviewed can count as a medium-sized enterprise with 80 staff during peak season, composed of both trained technicians and unskilled labourers.

The used AC business is very seasonal with substantially more and faster sales during the hot season in Metro Manila (February/March till end of May) than in the cooler or rainy months. During the low periods for sales of second hand units, maintenance and repair services become more important for most enterprises and individual technicians.

Most of the second hand shops or individual technician-retailers maintain several lines of business. Services offered include buying and selling of second hand ACs and its parts, repairing broken units or reassembling them to then sell and selling the scrap that cannot be reused anymore. Some also buy and sell parts only like compressors, for instance. Individual technician-retailers may work as service technician for a large AC company, e.g. Daikin or Koppel, and repair and sell used ACs on the side, obtained from clients whom they service as part of their primary occupation.

There was high agreement between interviewees that the competition in the second hand AC market is strong as profit margins are high. This forces especially business with employees to try and gain a competitive advantage. Individual technician-retailers tend to be less strategic, but rather opportunistic and flexible in adapting their mode of operations and services depending on whether a used or broken AC or parts of an AC are available. Some of them also simply sell broken or used ACs to the larger players in the second hand market (e.g. interview 12, March 3; interview 13, March 14, 2020).

In contrast to individual technicians, all enterprises interviewed try to find an advantage over competitors. These include the type of units and services offered, a warranty including free repair services (duration between 30 days and 6 months for used units), offers of discounts by not issuing receipts or when trading in old AC and general investments in stable customer relations. One respondent explicitly mentioned not giving warranty on inverter units as these are prone to malfunction due to their sensitive electronic boards (interview 24, March 11, 2020). In the Manila port area, hardly any difference between business models exists and shop owners do not seem to have a concrete strategy to attract customers.

Overall, the used AC market in Metro Manila is dominated by three to four larger players who have preferential access to second hand AC sources. Among these larger rivals, respect and friendly competition prevails, whereas stronger competition exists among the many smaller second hand shops and technician-retailer, also called “moonlighting technicians” (e.g. interview 13, March 14; interview 23, November 16; interview 30, October 29, 2020). Connections to middlemen and brokers are key in the procurement of used units. For green and social regulations, these findings imply that many livelihoods would be affected by interventions, but different degrees of impact could be expected on businesses in this market and individual technicians complementing their income. Larger changes to the market could be expected when targeting the suppliers and major players in the field, both in terms of environmental and social goals.

### Sources of ACs and Middlemen

The acquisition of used AC units (broken and functioning) presents the beginning of the second hand value chain. Understanding the sources of the units themselves as well as the role of middlemen or brokers who organize the supply to the second hand enterprises are important elements for any regulatory approach targeting the second hand market.

Sources of used ACs are primarily condominium buildings, hotels, office buildings and larger restaurants that renovate or are reconstructed or demolished, as reported by almost all respondents. A few retailers obtain used ACs at government auctions. Replacement of ACs in the homes of individual customers also happens with the technician taking the old AC for repairing and/or reselling, but this only constitutes a small market share. Previously, illegal imports of second hand ACs from Japan could be found in the Manila port area, in line with the tradition of Japanese surplus shops for all other kinds of goods. Upon the visit of the port area in February 2020, hardly any Japanese ACs could be found. Retailers gave the extra cost for a converter as a reason or preferred not to say what happened and whether they get any more ACs directly from the shipments (e.g. interview 12, March 3, 2020).

Brokers and partly family networks are key for the acquisition, as all interviewees mentioned. Brokers or middlemen have connections to demolition companies and the construction industry. Some larger retailers maintain connections to government property custodians, big firms, hotels and exporters in the export zones directly themselves. Retailers pay the middlemen for the used ACs. They also pay a small “finding fee” of 250–500P to tipsters (e.g. security guards, janitors, maintenance staff) who locate or bring used ACs to them (e.g. interviews 30, 32 and 34, October 29, 2020). Bribes play a significant role in the market in access to middlemen and their goods, to the construction industry or to win auctions. Larger players in the market are known to be able to pay substantial bribes: “They have the financial muscle to pay bribes and higher commissions to third-party brokers like engineering heads, supervisors and the like” (interview 13, March 14, 2020). Many respondents agreed that bribery or “tokens of gratitude” (e.g. interview 17; November 21, 2020) are standard in the business of used ACs.

The ties to middlemen are described as usually stable and based on reciprocity. In the port area, family networks tend to coincide with religious orientation of the shop owners (interviews 8, 9, 12; all March 3, 2020). A few larger second hand retailers also sell to technicians or shops in the provinces, turning into middlemen this way themselves. Additionally, a certain informal code of honour exists among all retailers: to pass on customers upon request, to remain in friendly competition and to not divulge the shadow system with brokers and bribery in detail to outsiders (interview 10, March 4, 2020). Here again, reciprocity presents a strong behavioural component of the market.

These findings on business characteristics and the business environment indicate an established value chain with large and small players with varying economic and social livelihood interests (market pull factors). The construction industry, brokers and retailers would all need to be addressed by future regulation (market push). The prices and profit margins of selling used ACs indicate what kind of incentive or compensation might be required for entrepreneurs to accept a regulation of the second hand market.

### Types and Prices of Used ACs

The types and prices of used ACs give insights into the profit margins as the core of prevalent business models. Understanding the market dynamics is required for proper targeting of regulatory measures to the actual models sold and to increase the chances for compliance.

In Metro Manila, the vast majority of shops and technician-retailers sell both window-type and split ACs, with window-type units slightly more common still. This reflects the overall market share that window-type units still have in the Philippines (62%, [12]. Units with smaller motors (1–1.5 Horsepower/HP) are common, whereas units with more than 2 HP cannot be found much in the second hand market. Used split-ACs with inverter technology are less common in the second hand market this far. A few businesses sell commercial units and compressors only. Compressors both within re-assembled units and sold as parts are sometimes re-branded or receive fake serial numbers (as stickers) to make them appear new or to be of a different brand. This kind of cheating practice is invisible to the lay customer and serves to increase the price and perceived quality of the product.

Stock generally sells fast, especially during the hot season with 1–3 days of stock turnover on average. In low season (non-COVID-19 pandemic times), 10–14 days are common till stock is sold. Stock visible in stores upon visit ranged from a handful of units to up to 30–40 units, or, in the case of retailers specialized on compressors, several shelves lined with compressors. It is not possible to give a specific number of stock and sales turnover as respondents were reluctant to share this information in detail. Due to the COVID-19 pandemic, sales numbers plummeted, for some retailers to 1–2 units sold per month. This already forced a few enterprises and technician-retailers out of business. Repair and maintenance services as well as re-assembly projects for later sale serve as a lifeline for business survival.

The prices for a used AC for the end consumer depend on the size, cooling capacity, brand and—to a lesser extent—the outward appearance of the unit. Prices of parts also vary and may in some instances be the most profitable part of the business. Interestingly, prices for complete used ACs largely converge, confirming that this is a well-established market in which demand, supply and competition regulate price levels (see Table 2).

**Table 2:** Average prices in the second hand market for ACs in Metro Manila (in Philippine peso)

Item	Acquisition (price paid to middle men or household)*	Sales price
AC 1HP (window-type)	3,000–4,000; broken unit: 500P	5,000–7,000
AC 1.5 HP (window-type)	3,000–5,000	6,000–8,000
AC 2 HP (window-type)	No data	8,500–9,000
AC 1 HP (split-type)	5,000–8,000	15,000
AC 1.5 HP (split-type)	No data	28,000
AC 2HP (split-type)	10,000–17,000	20,000–30,000
New compressor	25,000–32, 000	40,000 (with installation)
Used compressor	1,500–3,000	6,000–12,000

Source: Author’s interviews. *1,000 Philippine peso = 21 USD (currency conversion June 2021)

Overall, profits expected from the sales of second hand ACs range from 50 to 70% compared to the acquisition price, i.e. doubling or tripling investments for acquisition from middlemen. The exact profit depends on labour costs in the business, the number of AC purchases (bulk prices can be negotiated) and the investments and prices along the chain, including repair costs and scrap prices. Large players in the business will invest up to P 1 million (approx. 21,000 USD), for example, at government auctions and in middlemen, expecting profits to triple as whole units are sold to customers, parts left over are sold to other technicians and scrap sold to scrap dealers.

The enterprises that sell both new and used units indicated that between 15 and 35% of their final income derives from the second hand business, explaining why it is so attractive to almost all enterprises or technicians in the field. Individual technician-retailers may have a particularly profitable small-scale side-business, e.g. buying single used units from tenants in the building for P 1,000 and selling to new tenants for P 5,000 several times per year (interview 26, March 12, 2020).

In sum, the stability of and high returns in the second hand AC market indicate that regulatory interventions are likely to meet protest and/or counter-reactions by businesses to move into fully clandestine operations (market pull factors). As a large part of the system is happening informally, the prices do not reflect the technical value of the used units or the scrap, making the set-up of a formal collection system difficult—similar to what Streicher/Porte and Gerring [32] show for other used white goods in China. Alternative sources of income or the introduction of minimum quality standards for repaired units as a compromise may be necessary (market pull and push). Incentives or compensation schemes for a transition period (including business diversification, access to loans or credits) that cover 10–30% of incomes depending on the business size may be most successful.

### Customers and Purchase Motivations

The customer base and their reasoning for buying used ACs present the demand-side of the second hand AC market. Political interventions to change the market dynamics may also need to consider consumer motivations, especially if command-and-control instruments such as bans are not suitable or difficult to implement.

The type of customers buying used ACs are largely similar across Metro Manila. In the port area, retailers’ customers are mainly individuals and small shops or restaurants (e.g. interview 12, March 3; interview 20, November 21; interview 22. March 12, 2020). For the retailers and technician-retailers throughout Metro Manila, the share of individual customers is smaller than those of small enterprises. Restaurants, karaoke bars, small hotels, sub-contractors of fast-food chains, private companies and offices or mini supermarkets present the main customers, as reported by the vast majority of interviewees. Especially SME owners look for bargains to keep investment costs in their business low. Individuals from condominium buildings and individuals passing by or phoning in may also ask for the replacement or trade-in of a broken unit. Hardly any second hand shop or technician-retail can handle bulk orders of many units, but most can deal with an order of a few used units.

As the very poor cannot afford ACs, individual customers are mostly middle-income enterprises or middle class people looking for a bargain. Rarely, richer people ask for a used unit, as most retailers agreed. When it comes to individuals, retailers often find it hard to tell who these people are (e.g. interview 14, November 2020). Car ownership or expensive-looking clothing may give indications, some interviewees pointed out, but for all retailers, the proven ability to pay is more important than the type of customer. One interviewee mentioned that the middle class or rich people want new units in their homes, but prefer to buy used units for their businesses, saying that “used units will do” (interview 21, November 25, 2020).

Customers may also be other technicians or retailers from the provinces like Batangas, Laguna or Bulacan. Particularly, the larger retailers entertain a network of “suki”, i.e. loyal patrons that are either clients themselves or refer to final clients, other technicians and middlemen in the provinces (interview 13, March 14; interview 24, March 11; interview 29, March 12, 2020). The trade of compressors and other AC parts happens between technicians and retailer-technicians, also often based on relations of trust and reciprocity.

The uniting driver for the purchase of a used AC are the lower (or perceived lower) price and the speed and ease of availability of units. While well-known brands such as Carrier and Koppel are popular, the price and the cooling functionality override brand preferences—and usually also energy saving concerns: “Clients are not interested in energy rating. If it cools, it is ok with them” (interview 17, November 21, 2020). Customers seeking to make a bargain are likely to underestimate maintenance costs and remaining lifetime of a unit by focusing on upfront investment costs. The cost–benefit calculation may be favourable if a used unit costs P 6,000, has a warranty and lasts another 3 years without any repairs. It is not favourable if a more expensive refrigerant like R22 is used, remaining lifetime is short or maintenance costs are high, e.g. for an inverter unit. In those cases, new units from factory outlets outside of Manila are cheaper in the mid- to long term. For new units bought in regular stores, waiting times of several months are no exception during the hot season. Finally, using second hand appliances, repairing and reusing as long as possible presents the social norm in large parts of Filipino society, especially when affordability is a real constraint for the consumer.

In sum, the social implications of second hand market regulation for consumers need to be differentiated between individual customers or (lower) middle class families who otherwise cannot afford cooling and small service enterprises who could afford new units if they were offered energy-related loans or credits. The set-up of green credit lines and energy advisory services for SMEs may be helpful (market pull factors).

### Energy Efficiency and Refrigerant

Social and economic concerns of retailers and customers need to be balanced with energy and climate goals. Currently, energy efficiency and the refrigerant in the AC present secondary concerns to the vast majority of retailers and customers. In the second hand market, very few appliances still have the national energy efficiency label. If the label was still attached to the unit upon shop visits, it was unclear whether it was the original one or transferred from another AC. As some retailers also apply fake labels and serial numbers on (used) compressors, for example, caution regarding energy efficiency labels is unfortunately advisable for customers buying a used unit. Moreover, these re-labelling and to some extent re-branding of parts also cast some doubts whether specifications for the refrigerant contained are always correct. These findings confirm previous research on the effectiveness of labels and compliance in informal markets [33].

It is difficult to technically estimate how energy efficient the units arriving in the second hand market still are as respective preceding lifespans, use mode and maintenance are usually unclear. Most technicians agreed that ACs have a lifetime of 5–10 years in the Philippines and that repairing and reassembling them can add another few years—how many exactly is difficult to define. Maintenance and servicing of ACs (especially cleaning) play a large role for both the remaining energy efficiency and durability of used ACs.

The majority of used ACs are reported to arrive empty of refrigerant at the point of repair and resale, i.e. second hand shops or businesses. This implies that either units were completely empty upon deinstallation or venting/disposal of the refrigerant happens at the building site to be demolished or renovated building, or at the individual customer. If there is any refrigerant left, retailers or technicians tend to simply vent it. Few technicians try to capture the remaining refrigerant and dispose of it for recycling, saying they comply with regulations such as the Environmental Management Bureau Code on Air Condition Services [9]. According to the Code, ozone depleting substances (ODS) such as refrigerants in air conditioners shall be recovered with appropriate tools and stored in special containers to be surrendered to any dealer of ODS. Importers of ODS are required to provide adequate recycling facilities. If and exactly how this process happens in the second hand market remained unclear in the interviews.

Currently, a range of refrigerants are being used in the second hand market (R22, R32, R410a, R134a and R404a). As the refrigerant R22 is banned from 2021 onwards, ACs using this refrigerant were sold at a cheaper price in 2020 already. A common opinion among retailers and technician-retailers in the second hand market is that many used units with R22 will be available for another 10–15 years as the phase-out in the second hand market happens only gradually. The ban of refrigerants will therefore have a time lag in the informal second hand market. As Alam [1] reports, R22 for refills can still be bought across the Philippines. An effective import ban on R22 for refills will lead to a definite end of usage of this refrigerant,however, ACs made for R22 use may still be retrofitted for re-use with another refrigerant by some technicians.

As most customers focus on AC price and cooling capacity (see the “Types and Prices of Used ACs” section for prices), energy saving and energy efficiency do not influence their purchase decision, even if they are generally aware of the issue. The mental link between an energy efficient unit and savings on the electricity bill as an amortisation of a—higher—purchase price of an efficient unit is hardly made. In contrast, retailers and technician-retailers in the AC business have a higher awareness and knowledge about energy efficiency than customers, but they do not always have complete knowledge and/or convey information correctly to customers.

Those customers who are inquiring about energy saving sometimes also ask explicitly for an inverter AC. Here, two knowledge asymmetries exist: there are retailers/technician-retailers who advise clients to buy second hand inverter ACs for energy saving reasons (e.g. interview 12, March 3; interview 18, October 29; 2020) and others who explicitly do not do that, but tell clients to consider a new unit instead (interview 13, March 14, 2020; interview 29, October 29, 2020). The second knowledge asymmetry exists between retailers and customers: few clients are aware that inverter ACs require higher maintenance to be energy efficient and are less durable. They have seen commercials of new inverter ACs that advertise their higher energy savings or rely on word of mouth.

In the Philippines, inverter units are only more energy efficient than other split-ACs if they are maintained well and are running for a longer period of time. If they are turned on and off frequently or running for a short time, efficiency advantages can quickly evaporate. Moreover, the sensitive electric circuits in inverters produce need repairs or produce short-circuits quickly, e.g. due to dust accumulating or small animals such as lizards entering the unit. Consumers may end up with more overall costs than with a more traditional split-type AC, even if the electricity bill itself is lower (interview 9, March 9; interview 10; March 4, 2020). However, not all retailers are aware of these details either, especially those without any technical knowledge or training.

Few skilled technicians and retailers reflect critically about the overall benefit of used units in the AC market, saying, for instance: “It is better [for energy saving] to buy brand new units, but buying second hand or restored units sometimes makes sense to small establishment with low capitalizations, just for a start-up” (Interview 23, November 16, 2020). The majority of retailers in the market, however, do not think about energy efficiency questions in their daily business.

Retailers’ awareness levels of harmful ODS and refrigerants are generally lower than on oils and other hazardous waste. Awareness levels differ between trained technicians and other retailers or untrained technicians. Trained and skilled technicians tend to be aware of the various hazards. Some retailers and retailer-technicians know that the refrigerants are harmful to the environment and that they should be careful, but do not know why (interview 12, March 3; interview 14, November 16; interview 17, November 21, 2020). They either do not know what to do or lack the tools to handle remaining refrigerant appropriately. A few respondents mentioned lacking the tools to capture the refrigerant and do not know how to recycle it or do not worry at all about the refrigerant, oil, chemicals etc. Some shop owners are not aware at all and just pour remaining substances in the gutter (e.g. interview 18, October 29, 2020).

The compliance with existing codes and regulations is mixed. Many comply—or at least claim to comply—with government regulation on hazardous waste and the EMB code. Particularly the trained and certified technicians make an effort in this regard. Those retailers and retailer-technicians who do not comply believe that the government will never have enough technical inspectors and financial means to control (e.g. interview 21, November 25; interview 32, October 29; 2020). Among most interviewees, trust in government in general and in service delivery by sub-agencies is rather low. The vast majority of respondents favours not only a continued, but also more protected second hand market if the government in turn reliably offered more supportive services, e.g. micro-finance or financial incentives for energy efficient technology replacement.

**Fig. 1:**
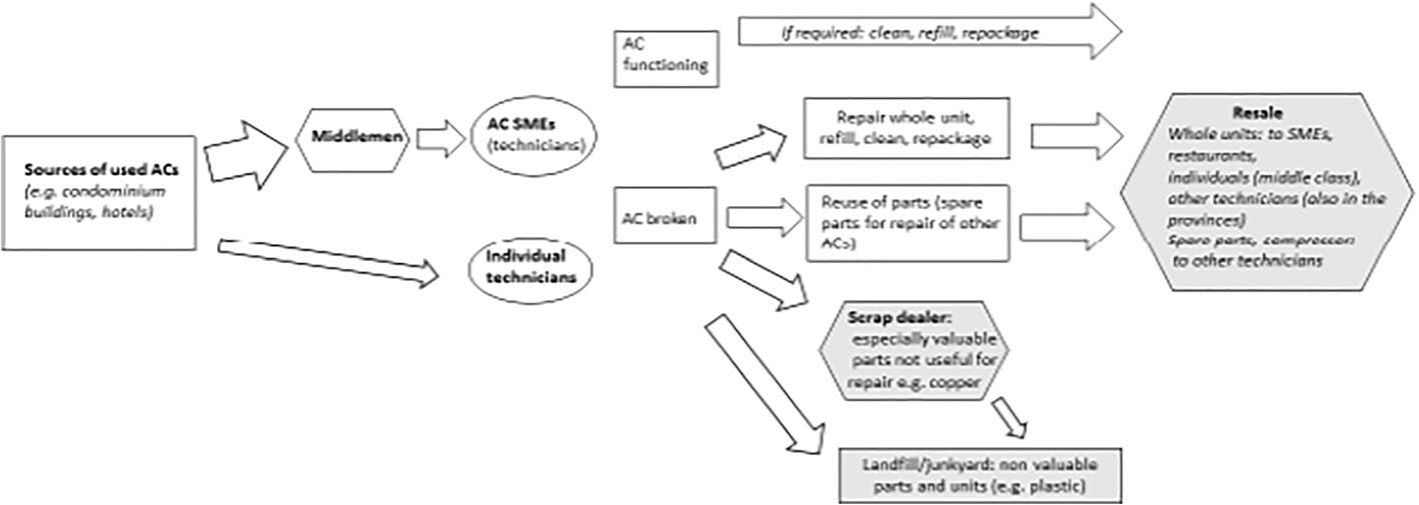
Schematic diagram of the second hand AC market in Metro Manila. Source: Author

For balancing energy and climate goals in the second hand AC market with material and social goals, these findings imply that a quick phase-out of inefficient ACs or at least efforts to improve energy efficiency, refrigerant disposal and repair standards are required. An established, easy to use system for ODS/refrigerant disposal is required that can become standard practice for retailers and technicians. Additionally, continued awareness raising for customers on cost-benefits including energy efficiency and climate harmful substances in used units as well as the limits of used inverter ACs is necessary (Fig. 1).

### Repairing and End Of life

Repair practices and quality are important to estimate environmental impacts and socioeconomic factors for the decision how to regulate the second hand market. Generally, AC technicians and second hand dealers interviewed repair and reuse all the materials possible. The technicians take out the copper wires, other metals such as nickel and useable parts including compressors to re-use and refurbish as much as possible. The plastic parts often cannot be reused and are taken to the landfill or junkyards close by. The majority of enterprises and technicians interviewed assemble and sell “new” units from various scrapped ACs, parts acquired over time and—less so—from a mix of used and new parts. Some enterprises also offer repair services for other appliances such as washing machines or rewind motors. A few technician-retailers are specialized in selling used compressors and other used parts of ACs. These results confirm Alam’s [1] findings on the end-of-life stage of ACs in other Philippine cities.

The type and brand of an AC matters for the extent to which it becomes a resource for reuse. For repair and reuse purposes, window-type room ACs are preferred as they are generally said to have more durable parts than split-type and inverter ACs (interview 9, March 9; interview 10, March 4; interview 13, March). Therefore, fewer inverter ACs are available in the scrap/reuse market. Panasonic ACs, especially inverters, can hardly be found at all—neither as second hand unit nor as scrap item that can dismantled and parts reused. New, first hand Panasonic ACs count as particularly durable, lasting approximately 10 years in primary use—“but after this time, the parts are so worn out that they cannot be reused at all” (interview 10, March 4, 2020). Some AC parts are interchangeable across specific brands such as Carrier and Koppel, making these models and their parts particularly popular among technicians and retailers.

The repair and reuse procedures can include some questionable practices. The restoration of old units or assembly from parts may include an “upgrading” to a higher class model, i.e. a class B unit is then sold as a class A model, or a used unit is sold as a completely new one. Some shops or technicians-retailers clean and polish the used ACs and wrap them in plastic to make them appear new. The customer usually cannot tell the difference. A few technicians-second hand dealers are specialized in selling used compressors and refurbishing compressors and/or ACs, either sell the compressor or a whole refurbished AC unit. This may include re-branding, putting false serial numbers and polishing/re-packaging the AC to sell at a higher price.

Once parts of ACs are deemed unusable or invaluable by retailers and technician-retailers, either scrap dealers pick up or receive the remaining parts, or the retailers bring them to junkyards or landfills. For the (informal) scrap dealers, copper and nickel as well as other metals present the most precious parts of an AC. These are usually dismantled or scraped out manually as even the larger enterprises lack the appropriate machinery for this kind of waste separation and recycling. Larger scrap dealers and wholesalers attend governmental auctions, for instance, in Valenzuela City and Mandaluyong City (both part of Metro Manila). When they buy scrap from technicians or second hand dealers, they sell the metals on to the smelting plants situated on the outskirts of Metro Manila.

Only few LGU have recycling sites for materials in Metro Manila and hardly any formally registered companies operate for ACs. There is only one accredited collection, transport and storage facility that can handle climate-damaging refrigerants. Additionally, some recycling sites in key industry areas and in the special economic zones with a more established system for hazardous waste exist. Existing drop-off stations for e-waste focus on smaller appliances this far, as do the public and civil society initiatives and campaigns active in the field this far. Here, a significant policy gap exists.

Among the retailers and technician interviewees, the idea of establishing an EPR system for ACs resonated well as long as it is run by private actors, as trust in governmental agencies to pay is low (interview 10, March 4; interview 22, March 12, 2020). For retailers and technicians, incentives or alternative sources of income via the integration in an EPR are likely more feasible than integrating informal recyclers and scrap dealers (market pull factors).

In terms of the material impact of the used AC market, our findings imply a high degree of informal circularity for compressors, metals and to some extent fans and valves. This finding questions the necessity of a short-term regulatory intervention in the market. However, repair and reuse practices are not environmentally friendly in those cases in which refrigerants are vented and used oils not disposed of properly. For plastic parts and inverter units, the material life in the second hand market ends more quickly and is likely harmful for the environment. For the end-of-life management of ACs, accessing and managing informal scrap dealers present a particular challenge. Moreover, more facilities and a more structured system for recycling of materials and refrigerants are required.

## Conclusion and Policy Implications

The balancing of energy, climate, materials resource and social aspects in the second hand AC market requires the appropriate timing and combination of measures. Overall, the improvement of second hand AC quality, including repair and reuse of ACs, to foster more positive development effects in line with a circular economy approach is desirable. The exact point in time in the lifecycle of an AC at which environmental and social costs and benefits are on par is difficult to identify without deeper technical analyses of AC units in the Philippine second market.

The analysis has shown that short- to mid-term regulations and interventions are more appropriate than postponing to the long term or foregoing any regulation. From an energy and climate perspective, phasing-out used ACs as quickly as possible is desirable; from a social and waste management perspective, allowing for a short prolongation of an AC life by approximately 1–3 years is more attractive than no affordable ACs and lost jobs. Ideally, all very old AC units, for example, those entering the used AC market for a second time or broken units with more than 3–5 years added lifetime after primary use (i.e. 8–10 years), should be phased out quickly.

The following policy options could be explored, categorized along the well-known “stick-carrot-tanmbourine” categories of energy policy:

- Ban second hand market (“stick”): not useful because (a) resource waste/waste problem likely to increase as no complete, professionally organized disposal/recycling system in place yet, (b) this would deprive many Filipinos of access to an affordable cooling device; (c) employment/livelihoods in the market would be quite strongly affected as a substantial part (min. 10–30%)of income for both individual technicians and SMEs active in the sector would be cut; (d) second hand market would only become completely clandestine then.
- Regulate construction industry and building demolition (“stick”): Change building code/demolition and waste regulation to (a) enforce AC and refrigerant disposal as hazardous waste, (b) set fines for middlemen who do not comply, and (c) *develop and set clear conditions which used AC units can enter the second hand market* (e.g. based on age, type and horsepower of unit, refrigerant) and which used ACs are banned from any reuse. For example, it could be debated whether units with a lot of horsepower or suitable for large rooms need to be available at affordable prices (as poorer households usually do not have many or very large rooms that need cooling).
- Set-up of professional reuse and recycling scheme that integrates the second hand market (“carrot and tambourine”, see Streicher-Porte/Gerring [32] for a similar suggestion for white goods in China): This would require more specific electronic waste regulation and the development of an EPR scheme, possibly complemented by a used AC rebate scheme for consumers, technicians and scrap dealers to hand in least efficient ACs. Technicians and retailer-technicians signalled general openness if an EPR scheme was privately run (low trust towards government agencies).
- Gradual set-up of repair standards for consumer and technician information (“tambourine”), as suggested in the WEEE literature for other white goods (e.g. [23]: A gradual process with a voluntary process to ensure buy-in of all stakeholders is advisable. Starting points could be a competition and public award for the most energy efficient, climate friendly, repaired AC or designing a behaviour change intervention aimed at technicians and technician-retailers’ pride, trust and system of reciprocity to self-establish a repair standard.
- Establish green credit lines and energy advisory services for SMEs/small service industry (“carrot”) to incentivize and enable these enterprises to buy new ACs and refrain from perceived bargains of used ACs, as especially owners of small restaurants, bars and other SMEs buy second hand ACs in the Philippines.

## Data Availability

Primary qualitative data collected during research stays in Manila 2020 with the help of Ramon Ortiz, a local research assistant.

## Annex

Table 3

**Table 3:** List of institutions interviewed:

Abbreviation	Full name
DoE	Department of Energy Philippines (several interviewees)
DENR	Department of Environment and Natural Resources
NEDA	National Economic and Development Authority Philippines
GIZ	Deutsche Gesellschaft für Internationale Zusammenarbeit
UNIDO	United Nations Industrial Development Organization
RACTAP	Refrigeration and Air Conditioning Technician Association of the Philippines
CCI-Hub	Cold Chain Innovation Hub
PIDS	Philippine Institute of Development Studies
UP	University of the Philippines

For reasons of anonymity, only the institutions, no individual names, can be listed. Planned interviews with the Department of Trade and Industry, TESDA and the Valenzuela City government Waste unit had to be cancelled due to the outbreak of the COVID-19 pandemic
